# Cross-sectional data on stablecoin characteristics

**DOI:** 10.12688/f1000research.126298.1

**Published:** 2022-10-17

**Authors:** Katarzyna Włosik, Blanka Łęt, Konrad Sobański, Wojciech Świder

**Affiliations:** 1 Department of Investment and Financial Markets, Poznań University of Economics and Business, Al. Niepodległości 10, 61-875 Poznań, Poland; 2 Department of Applied Mathematics, Poznań University of Economics and Business, Al. Niepodległości 10, 61-875 Poznań, Poland; 3 Department of International Finance, Poznań University of Economics and Business, Al. Niepodległości 10, 61-875 Poznań, Poland; 4 Department of Public Finance, Poznań University of Economics and Business, Al. Niepodległości 10, 61-875 Poznań, Poland

**Keywords:** Stablecoin, cryptocurrency, whitepaper, exchanges, market cap, collateral type

## Abstract

The article presents a dataset on the characteristics of stablecoins. Stablecoins represent a relatively young but increasingly important branch of the cryptocurrency market. Although they all share the same goal of maintaining a stable value in the digital market, they form a highly heterogeneous group. They differ in terms of collateral and stabilization mechanism, peg, availability of the technical documentation, presence on crypto exchanges or age. The dataset is cross-sectional and was created based on internet research. Individual information was collected from websites of the stablecoin projects and a crypto-data aggregator, and to a lesser extent from other auxiliary sources (websites related to finance and cryptocurrencies). The dataset is unique as there are no publicly available databases encompassing the features of stablecoins. It can be used in all stablecoin-related analyses to characterise the examined coins and to investigate the relationship between cryptocurrency market developments and stablecoin features.

## Introduction

Stablecoins can be defined as digital units of value that, through special stabilisation mechanisms, are supposed to maintain a stable value in relation to a selected benchmark – one or more traditional currencies or other types of assets, including crypto assets. ^1^ Stablecoins are drawing the attention of policymakers due to their dynamic growth, the increasing number of use cases and the risks they may pose to financial stability. ^1^ The stablecoin market is also appealing to investors and researchers. Nevertheless, very few papers explore the importance of stablecoin features. The work of García *et al*., ^2^ who consider stablecoin features when developing a framework for risk assessment, is among the exceptions. Jarno and Kołodziejczyk ^3^ analyse the volatility of stablecoins with respect to the type of these digital assets. It is worth noting that while all stablecoins aim to maintain a stable value, they differ in many aspects. This fact creates research opportunities.

The dataset presented in this Data Note combines multiple data sources on individual stablecoins to form a unique database on the stablecoin market (on features of stablecoins). It can be useful for researchers and practitioners interested in the functioning and development of the cryptocurrency market. The dataset might be used for cross-sectional comparative studies on the stablecoin market. This branch of the cryptocurrency market is relatively young and there are no publicly available databases encompassing the features of these digital assets. The dataset can be applied in all stablecoin-related analyses to characterise the examined coins and investigate the relationship between cryptocurrency market developments and stablecoin features without the need to dedicate efforts to time-consuming studies of individual stablecoins.

## Methods

The data was collected via internet research. The list of stablecoins was compiled from information available on the website of CoinMarketCap – the aggregator of cryptocurrency data which is frequently used in research (see for example Refs. 4– 7). Then, data on these stablecoins was collected from various sources – websites of the stablecoin projects, CoinMarketCap, and, to a limited extent, other websites.

Out of the group of 98 stablecoins that were listed by CoinMarketCap (as of 8 May 2022) the data was collected for 30 of them. A coin was excluded from the analysis if:

• it was launched after 1 July 2021,
• there was virtually no information on this stablecoin and the project’s website was not working when the study took place (e.g. Xaurum.org),
• it was misclassified as a stablecoin – such cases were determined based on the price charts provided by CoinMarketCap and also the analysis of the projects’ websites (e.g. Reserve Rights – RSR, was classified as a stablecoin when the study took place, however the price chart showed great variability ^8^ and the website of the project informed that RSR was a governance token supporting the functioning of a stablecoin developed within the same protocol ^9^^,^^10^),
• its operation has been suspended (e.g. DigixDAO ^11^).

The information used to create the database came from several sources:

• the CoinMarketCap.com website (list of stablecoins, tickers, approximated start date, market capitalisation, number of crypto exchanges listing a particular stablecoin, number of trading pairs in which a particular stablecoin is included),
• websites of the analysed stablecoin projects (type of stablecoin, availability of whitepaper/documentation, peg),
• other websites (other relevant information, e.g. link to any crypto exchange).

Due to the large number of websites, their addresses are provided in the dataset file. Each data point is accompanied there by a source.

## Data description

The dataset (see *Underlying data* ^12^) includes 30 stablecoins: Tether, USD Coin, Binance USD, Terra USD, Dai, True USD, Pax Dollar, Neutrino USD, Frax, HUSD, Origin Dollar, Gemini Dollar, Stasis Euro, sUSD, Celo Dollar, Qcash, Vai, Steem Dollars, mStable USD, USDK, Rupiah Token, EOSDT, CryptoFranc, NuBits, Italian Lira, Fei, Liquity USD, XSGD, TerraKRW, Basis Cash.

The database contains 14 features characterising each of the above-mentioned stablecoins. The features were selected to show different aspects of stablecoins, including their technical properties, connections to different trading venues and properties that may affect their attractiveness, credibility and availability for market participants. The description of these characteristics is provided in Table 1.

**Table 1.** Characteristics of stablecoins included in the database and their description.

Characteristic	Description
1. Link to any crypto exchange	Stablecoins can be bought or sold on various trading venues – so-called crypto exchanges. In the case of ten stablecoins that are included in the dataset, there are some relationships between the stablecoin project and a crypto exchange. As indicated by Rosa and Pareschi, ^13^ there are co-ownership and co-administrative relationships between the issuer of Tether and Bitfinex. Pax dollar and the crypto exchange itBit are linked via the company Paxos. ^14^ The issuer of HUSD is a strategic partner of Huobi Global. ^15^ USD Coin is related to Coinbase, ^16^ Binance USD to Binance, ^17^ and TerraUSD was launched in cooperation with Bittrex Global. ^18^ OKEx launched USDK ^19^ and Gemini – Gemini dollar. ^20^ Decentralized Forex (DeFo) is a decentralized exchange that is an extension of the Neutrino protocol on which Neutrino USD is based. ^21^^,^^22^ And sUSD is linked to Kwenta. ^23^ In the database, this is a dummy variable (1 – stablecoin is linked to a crypto exchange; 0 – stablecoin is not linked to any crypto exchange).
2. Peg to the USD (United States dollar)	Prices of stablecoins are pegged to selected benchmarks. This variable shows whether a stablecoin is pegged to the USD, as the USD is one of the most frequently used benchmarks in the stablecoin universe and it also dominates in the dataset. The remaining stablecoins that are included in the dataset are pegged to the euro (Stasis Euro), the Chinese yuan (Qcash), the Indonesian rupiah (Rupiah Token), the Swiss franc (CryptoFranc), the Singapore dollar (XSGD), and the South Korean won (TerraKRW). For one stablecoin – Italian Lira, there is no official information on the peg. In the database, this is a dummy variable (1 – stablecoin is pegged to the USD; 0 – stablecoin is not pegged to the USD).
3. Availability of a whitepaper or documentation on the stablecoin website	During the data collection phase, it was verified whether a whitepaper or technical documentation for the stablecoin project was available on its website. It is worth noting that there are websites that collect whitepapers related to cryptocurrency projects. However, it is not always possible to verify by whom and when the document was provided. This is why only the official websites of the projects have been considered. In the database, this is a dummy variable (1 – a whitepaper/ documentation is available on the stablecoin website; 0 – a whitepaper/documentation is not available on the stablecoin website).
4. Approximated age of the analysed stablecoins (in days)	Stablecoins differ also in terms of age. For many stablecoins, the exact date of inception has not been provided by their issuers. Therefore, the starting data point from the CoinMarketCap website is used as a proxy for the inception date. The age is expressed as the number of days between the inception date and 6 May 2022.
5. Classes of stablecoins based on their age	Stablecoins can be grouped by age (based on variable 4). They are classified as ‘old’ if they were launched at least 1,000 days before 6 May 2022. They are considered ‘young’ if the number of days is 500 or less. Otherwise, they are included in the ‘middle’ category.
6. Presence of stablecoins on major exchanges	Stablecoins can be traded on crypto exchanges. However, these digital assets differ significantly in terms of the number of exchanges that list them. A major exchange is defined here as one of the top ten crypto exchanges by share in total trading volume according to the CoinMarketCap ranking. This variable shows the number of major exchanges that list a given stablecoin.
7. Classes of stablecoins based on their presence on major exchanges	This variable presents classes of stablecoins based on their presence on major exchanges (based on variable 6). Some stablecoins included in the dataset were not listed by any of these exchanges (class ‘none’). If a stablecoin was listed by only one major exchange, its presence is ‘limited’. If it was listed by two to ten major exchanges, its presence is classified as ‘significant’.
8. Number of crypto exchanges listing a particular stablecoin	Stablecoins also differ in terms of the total number of exchanges that list them, which is reflected by this variable.
9. Classes of stablecoins based on the number of crypto exchanges listing a particular stablecoin	Based on the general number of crypto exchanges that list a particular stablecoin (based on variable 8), these cryptocurrencies can be divided into groups. The number of exchanges is considered ‘large’ when it is equal to or exceeds 50. This number is considered ‘moderate’ if it is between 11 and 49. Otherwise, it is considered ‘small’.
10. Number of trading pairs containing a particular stablecoin	Even on a single exchange, a particular stablecoin can be included in multiple trading pairs (e.g. BTC/USDT – Bitcoin to Tether). This variable reports the approximated number of such pairs for each stablecoin.
11. Classes of stablecoins based on the number of trading pairs containing a particular stablecoin	Based on the number of trading pairs containing a particular stablecoin (based on variable 10), these digital assets are divided into three groups. The number of pairs is considered ‘large’ when it is at least 200. The number is considered ‘moderate’ when it is between 31 and 199, and ‘small’ – when it is up to 30.
12. Stablecoin type	Stablecoins differ in type. They can be backed by assets – traditional assets, usually fiat currencies or assets denominated in fiat currencies, and digital assets – cryptocurrencies and other digital tokens. There are also algorithmic stablecoins. These are governed by an algorithm and smart contracts that regulate their supply and aim to ensure price stability. The number of stablecoin units in circulation is reduced when the price falls below peg, and increases when the price exceeds peg. ^24^ A systematic discussion of stablecoin design characteristics can be found in Ref. 25. In the dataset, four groups of stablecoins are distinguished based on their type. The first group contains stablecoins that are backed by fiat currencies and/or assets denominated in fiat currencies. The second group – stablecoins that are at least partially crypto-backed. The evolution of the cryptocurrency market has led to the emergence of hybrid stablecoins, which combine features of different types. Therefore, this category contains stablecoins that are either collateralised by crypto assets or are both collateralised by these assets and use an algorithm at the same time. It is worth noting, however, that the collateral is declared by the developer. The third group contains algorithmic stablecoins. For one coin – Italian Lira, no information is available.
13. Median market capitalisation in USD	This variable reflects the median market capitalisation for each stablecoin included in the dataset. The median is calculated over the period from 23 November 2021 to 6 May 2022 based on daily data.
14. Median trade volume in USD	The median trade volume for each stablecoin from the dataset is calculated over the period from 23 November 2021 to 6 May 2022 based on daily data. It needs to be underlined, however, that when analysing trade volume that is aggregated across exchanges, one needs to be aware of its limitations. This variable is criticised in the literature for being biased and inflated (for a discussion see Ref. 26).

## Data availability

### Underlying data

Mendeley Data: Cross-sectional data on stablecoin characteristics. https://doi.org/10.17632/v4pgsyn6dr.1. ^12^

This project contains the following underlying data:

- Dataset.xlsx

Data are available under the terms of the Creative Commons Attribution 4.0 International license (CC-BY 4.0).

## References

[1] Adachi M Pereira Da Silva PB Born A : Stablecoins’ role in crypto and beyond: functions, risks and policy. European Central Bank;2022. (accessed 14 September 2022). Reference Source

[2] García A Lands B Yanchus D : Stablecoin assessment framework.Bank of Canada Staff Discussion Paper, 2021, No. 2021-6, Bank of Canada, Ottawa.

[3] Jarno K Kołodziejczyk H : Does the Design of Stablecoins Impact Their Volatility? *J. Risk Fin. Manag.* 2021;14(42). 10.3390/jrfm14020042

[4] Kristoufek L : Tethered, or Untethered? On the interplay between stablecoins and major cryptoassets. *Financ. Res. Lett.* 2021;43:101991. 10.1016/j.frl.2021.101991

[5] Assaf A Bhandari A Charif H : Multivariate long memory structure in the cryptocurrency market: The impact of COVID-19. *Int. Rev. Financ. Anal.* 2022;82:102132. 10.1016/j.irfa.2022.102132

[6] Long H Zaremba A Demir E : Seasonality in the Cross-Section of Cryptocurrency Returns. *Financ. Res. Lett.* 2020;35:101566. 10.1016/j.frl.2020.101566

[7] Meyer A Ante L : Effects of initial coin offering characteristics on cross-listing returns. *Digital Finance.* 2020;2:259–283. 10.1007/s42521-020-00025-z

[8] CoinMarketCap: Reserve Rights. 2022. (accessed 8 May 2022). Reference Source

[9] Reserve: Introduction to the Reserve protocol.(accessed 8 May 2022). Reference Source

[10] Reserve Rights (RSR): Introduction to the Reserve protocol.(accessed 8 May 2022). Reference Source

[11] Digix: Customer Notice.(accessed 8 May 2022). Reference Source

[12] Włosik K Łęt B Sobański K : Cross-sectional data on stablecoin characteristics, Mendeley Data, V1, 2022. [Dataset]. 10.17632/v4pgsyn6dr.1 PMC9898686 36761836

[13] Rosa G Pareschi R : Tether: A Study on Bubble-Networks. *Front. Blockchain.* 2021;4:686484. 10.3389/fbloc.2021.686484

[14] Paxos: Pax Dollar (USDP): White Paper. 2021. (accessed 8 July 2022). Reference Source

[15] Huobi Global: The Safe and Secure Stablecoin. Frequently Asked Questions. Who is the issuer of HUSD? 2022. (accessed 8 July 2022). Reference Source

[16] Centre: Introducing USD COIN: A stablecoin brought to you by Circle and Coinbase. 2022. (accessed 8 July 2022). Reference Source

[17] Paxos: Binance USD (BUSD). 2022. (accessed 8 July 2022). Reference Source

[18] Kwon D : Announcing TerraUSD (UST) – the Interchain Stablecoin. 2020. (accessed 8 July 2022). Reference Source

[19] Perryman E : What is the USDK stablecoin? 2019. (accessed 8 July 2022). Reference Source

[20] Gemini: Gemini dollar. 2022. (accessed 8 July 2022). Reference Source

[21] Neutrino: Decentralized Forex. Trade national currencies on a decentralized exchange. 2022. (accessed 8 July 2022). Reference Source

[22] Neutrino: USDN. 2022. (accessed 8 July 2022). Reference Source

[23] Kwenta: FAQ. 2022. (accessed 8 July 2022). Reference Source

[24] Gemini: What Are Stablecoins? 2022. (accessed 8 July 2022). Reference Source

[25] Moin A Sekniqi K Sirer EG : SoK: A Classification Framework for Stablecoin Designs. Bonneau J Heninger N , editors. *Financial Cryptography and Data Security. FC 2020. Lecture Notes in Computer Science.* Cham: Springer;2020;12059.

[26] Alexander C Dakos M : A critical investigation of cryptocurrency data and analysis. *Quant. Finance.* 2020;20(2):173–188. 10.1080/14697688.2019.1641347

