# Supplementary material for: Cross-sectional data on stablecoin characteristics
Source: F1000Res. 2022 Oct 17;11:1188. [Version 1] doi: 10.12688/f1000research.126298.1 (PMC9898686; doi:10.12688/f1000research.126298.1)
Supplement: Peer review history

# Peer review: Reviewer response for version 1

**DOI:** 10.5256/f1000research.138695.r154508

**Published:** 2023-02-03

**Authors:** Rekha Pillai

**Affiliations:** 1 College of Business Administration, Ajman University, Ajman, United Arab Emirates

The article is novel. The rationale for creating the aforesaid data set is clearly outlined. The authors have collected Individual information from websites of the stablecoin projects and a crypto-data aggregator and they have clearly mentioned about the limited availability of stablecoin related information available on the public domain. It can be considered as an exploratory study as it unearths the stable coin dimensions, a less researched topic but one of high significance.  

 Future studies can build on the same and this is the main contribution of the paper. The data set is clearly presented in a useable and accessible format. It is clearly evident that no other cross- sectional studies of a similar nature has been conducted till date. However, as a suggestion, you may also justify the rationale behind why only 30 stable coins were selected, although the attempt is highly appreciated. You have clearly highlighted the rationale in excluding certain stable coins but you may elaborate on the total available, ones included and those excluded for providing a comprehensive picture. 

 As a recommendation to improve the paper, a brief literature review in a tabular form which only contains author names, year and key findings can add value. The paper may include a concluding paragraph, wrapping up the study with some future research/practical implications. Limitations of the study can be highlighted and suggest potential use of aforesaid data collected as recommendations for future research. 

 Finally thank you for giving this opportunity to review the paper and I hope the comments will be taken positively.

Are sufficient details of methods and materials provided to allow replication by others?

Yes

Is the rationale for creating the dataset(s) clearly described?

Yes

Are the datasets clearly presented in a useable and accessible format?

Yes

Are the protocols appropriate and is the work technically sound?

Yes

Reviewer Expertise:

Corporate governance, cryptocurrency, firm performance, behavioral finance

I confirm that I have read this submission and believe that I have an appropriate level of expertise to confirm that it is of an acceptable scientific standard.

# Peer review: Reviewer response for version 1

**DOI:** 10.5256/f1000research.138695.r156525

**Published:** 2022-12-05

**Authors:** Sergio Luis Náñez Alonso

**Affiliations:** 1 DEKIS Research Group, Department of Economics, Catholic University of Ávila, Avila, Spain

The data note under review presents a brief introduction to the characterization of digital currencies called Stablecoins. This has allowed the authors to build up a novel database on stablecoins, mainly by searching the Internet. It is therefore a brief scientific review of the current state of the art on stablecoins, proposing a database that can be used by other researchers in their studies. It is in this last point that the value of the study lies.

 After reviewing the data note, it can be qualified as highly original, given that there are no other cross-sectional databases available for consultation by potential cryptocurrency researchers. This means that the contribution to scholarship is also high.  Regarding the structure, the data note under evaluation is of the short-paper type, so the introduction is sufficient.  

 There are a few issues that should be improved by the authors:

 In the methodology section, the authors should refer to previous database generation studies with their limitations.

 In the data description section, the authors should indicate a valid reason why only 30 Stablecoins were selected. In other words, originality in the attempt to construct this database is appreciated. The methodology details the criteria for selecting the sample of 30 stablecoins based on the information that appears in CoinMarketCap, the websites of the stablecoins themselves and other websites (at this point, they could mention some, perhaps including references). I understand that of the 98 listed on CoinMarketCap as of May 2022, many were excluded (down to 30) for the reasons stated. I don't know if Terra USD is no longer classified as a stablecoin after the crash that month (it dropped 40% in value). Do you guys consider keeping it in the sample? If so, I would like you to explain.

 I find table 1 very interesting as it raises 14 characteristics (a sufficient number) and a description of these. It is a research note that adds value to academic research on this topic. I recommend, however, to expand the references, either in the text or in Table 1, as there are many publications on stablecoins, in order to characterize stablecoins with previous studies and authors.

 Finally, I thank you for inviting me to review this data note. I found it relevant and interesting.

Are sufficient details of methods and materials provided to allow replication by others?

Yes

Is the rationale for creating the dataset(s) clearly described?

Yes

Are the datasets clearly presented in a useable and accessible format?

Yes

Are the protocols appropriate and is the work technically sound?

Yes

Reviewer Expertise:

Digital Economy, Decentralised Finance, Central Bank digital Currency

I confirm that I have read this submission and believe that I have an appropriate level of expertise to confirm that it is of an acceptable scientific standard.
